# Identification of a novel homozygous variant in the alkaline phosphate (*ALPL*) gene associated with hypophosphatasia

**DOI:** 10.1002/ccr3.2962

**Published:** 2020-06-21

**Authors:** Atil Bisgin, Ibrahim Boga, Cihan Cetin, Selim Buyukkurt

**Affiliations:** ^1^ Medical Genetics Department of Balcali Clinics and Hospital Faculty of Medicine Cukurova University Adana Turkey; ^2^ Cukurova University AGENTEM (Adana Genetic Diseases Diagnosis and Treatment Center) Adana Turkey; ^3^ Obstetrics and Gynecology Department of School of Medicine Bahcesehir University Istanbul Turkey; ^4^ Perinatology Unit Obstetrics And Gynecology Department of Balcali Clinics and Hospital Faculty of Medicine Cukurova University Adana Turkey

**Keywords:** genetic counseling, hypophosphatasia, prenatal diagnosis

## Abstract

The lack of awareness of patient risk factors, failure to obtain adequate family history, was discussed by clinical experience in prenatal testing of hypophosphatasia with a novel variant in the ALPL gene identified in the index case of the family.

## Graphical abstract

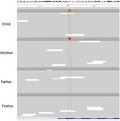

## 1 INTRODUCTION

Hypophosphatasia is a rare genetic disease characterized by a low tissue nonspecific alkaline phosphatase activity (TNSALP), due to the *ALPL* gene (OMIM# 241 500) variant. The prevalence of severe forms of this disease has been estimated at 1/100 000. Disease diagnosis is based on laboratory assays and DNA sequencing of the ALPL gene. Depending on the age of the patients when they are diagnosed, six clinical forms are currently recognized: perinatal (lethal), perinatal benign, infantile, childhood, adult, and odontohypophosphatasia.^4^, ^5^, ^10^ This degree of clinical heterogeneity significantly increases the importance of genetic counseling due to multiple factors including a complicated variable inheritance pattern (autosomal dominant or autosomal recessive); the existence of an uncommon prenatal benign form; and the incomplete penetrance of the disease trait.^1^, ^3^, ^4^, ^9^ An infantile form of hypophosphatasia with severe convulsions and rickets/osteomalacia findings is reported.

## 2 CLINICAL EXAMINATIONS AND CASE PRESENTATION

An eight‐week pregnant woman who was referred to our Medical Genetics Clinics had a 2‐year‐old boy suffering from severe convulsions and rickets/osteomalacia without any confirmed diagnosis. At the end of a detailed examination with baseline laboratories to obtain the underlying cause of rickets to were notable for calcium, phosphorus, and parathyroid hormone, poor linear growth, mild limb bowing, and radiographic rickets by a complete skeletal survey that demonstrates metaphyseal irregularities in the long bones and costochondral areas. However, the mother was also clinically followed up confirming that she had no other findings. During the history taking, we observed that the pregnant woman in this report did not receive full prenatal care and the imaging; even the limited ultrasound had never been completed in her previous pregnancy and she never had any genetic counseling. Thus, when we take the family history into an account, a presumptive diagnosis of hypophosphatasia was achieved. For the molecular diagnosis of the child, an *ALPL* whole gene sequence of all exons and introns was performed by next‐generation sequencing (NGS) (Illumina MiSeq Systems, California, USA). The *ALPL* gene exists as a single copy in the haploid genome at chromosome 1 and locus to 1p36.1‐p34 and contains 12 exons distributed over more than 50 kb.^2^, ^8^ For prenatal diagnosis, chorionic villus samplings (CVS) were obtained and genetic analysis was carried out by the same methods.^7^

**Figure 1:**
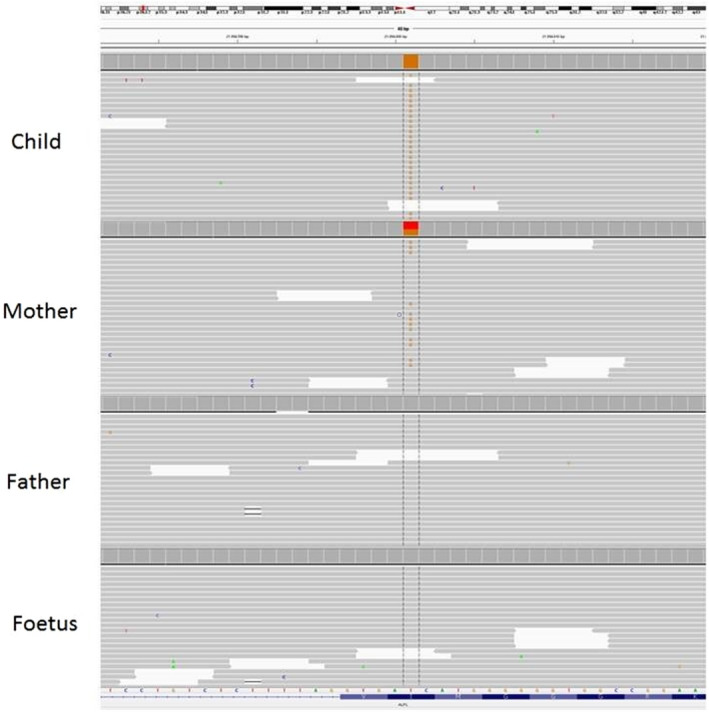
Sequence view of the ALPL gene of family: The first line is child's, the second line is mother's, the third line is father's, and the fourth line is fetus's ALPL gene sequence view

A novel p.I218S (c.653T > G) (NM_000478.6) homozygous variant was detected in the child as a result of the genetic analysis. Since there was another identified variant in the same position and had been reported in The Single Nucleotide Polymorphism Database (dbSNP), in silico analysis by CADD, MutationTaster, SIFT, and FATHMM was performed, which classified the variant as likely pathogenic due to the ACMG criteria in which we have PM1, PM2, PP2, and PP3.^6^ Thus, due to the results obtained from genetic testing both parents were analyzed for the same variant to identify their carrier status. The identical variant in heterozygotic form was observed in the mother, which defines her as a carrier; the father, however, had no ALPL variant (Figure 1). After a careful family counseling, to clarify the loss of heterozygosity or any deletion in the ALPL gene and also for the paternity, MLPA (multiplex ligation‐dependent probe amplification) testing and SNP array had been offered but the couple refused for further testings. Even though any follow‐up tests could not be performed, according to the results obtained and the genetic counseling, the family decided to have a prenatal testing for the hypophosphatasia. Thus, we performed prenatal cytogenetic and molecular testing with permission from the family to analyze whether the fetus had the variant from the obtained CVS (chorionic villus sampling) during the 10th week of pregnancy, but no variant in the fetus had been detected and also the karyotype analysis revealed no other abnormalities.

All procedures performed in this study were in accordance with the ethical standards of the institutional ethical and national research committee and with the Helsinki Declaration.

## 3 DISCUSSION

This case‐based report showed both the importance of clinical genetic assessments and genetic counseling for rare disease diagnoses and a novel p.I218S (c.653T > G) homozygous variant in the *ALPL* gene, which allowed for the rapid specific testing of a fetal sibling of the affected proband.

It is no surprise that modern genetic testing strategies have already been shaping the practice of medicine and more specifically the practice of genetic counseling in terms of how we practice and the way patients consume information. Careful counseling with the awareness of nonstandard syndromes/diseases has the potential to aid in at‐risk patient identification, assist in performing a differential diagnosis, and improve efficiency in collecting the medical history and risk assessment.

The use of molecular diagnostics is on the rise due to an increased awareness of rare diseases by both healthcare specialists and their patients. As such, more clinical geneticists are needed to diagnose and assess these patients and their families for prenatal testing, diagnosis, and clinical follow‐ups with proper genetic counseling.

To sum up, here we report the importance of genetic counseling even with challenges to integration and propose all the diagnostic applications that can shape the daily clinical practice.

## CONFLICT OF INTEREST

None declared.

## AUTHOR CONTRIBUTIONS

AB: conceived and designed the experiments. CC and SB: collected the biological materials and referred patients. IB: performed the experiments. AB and IB: performed the analysis, interpreted the data, and wrote the paper.

## CONSENT

Written consent was obtained from all patients.
